# SARS-CoV2 billion-compound docking

**DOI:** 10.1038/s41597-023-01984-9

**Published:** 2023-03-28

**Authors:** David M. Rogers, Rupesh Agarwal, Josh V. Vermaas, Micholas Dean Smith, Rajitha T. Rajeshwar, Connor Cooper, Ada Sedova, Swen Boehm, Matthew Baker, Jens Glaser, Jeremy C. Smith

**Affiliations:** 1 Computing and Computational Sciences Directorate, Oak Ridge National Laboratory, Oak Ridge, TN 37831 USA; 2 UT/ORNL Center for Molecular Biophysics, Oak Ridge National Laboratory, Oak Ridge, TN 37831 USA; 3 Department of Biochemistry and Cellular and Molecular Biology, The University of Tennessee, Knoxville, Knoxville, TN 37996 USA; 4 MSU-DOE Plant Research Laboratory, Michigan State University, East Lansing, MI 48824 USA; 5 Biological Sciences Division, Oak Ridge National Laboratory, Oak Ridge, TN 37831 USA

**Keywords:** Virtual screening, Computational chemistry, Structure prediction, Viral infection

## Abstract

This dataset contains ligand conformations and docking scores for 1.4 billion molecules docked against 6 structural targets from SARS-CoV2, representing 5 unique proteins: MPro, NSP15, PLPro, RDRP, and the Spike protein. Docking was carried out using the AutoDock-GPU platform on the Summit supercomputer and Google Cloud. The docking procedure employed the Solis Wets search method to generate 20 independent ligand binding poses per compound. Each compound geometry was scored using the AutoDock free energy estimate, and rescored using RFScore v3 and DUD-E machine-learned rescoring models. Input protein structures are included, suitable for use by AutoDock-GPU and other docking programs. As the result of an exceptionally large docking campaign, this dataset represents a valuable resource for discovering trends across small molecule and protein binding sites, training AI models, and comparing to inhibitor compounds targeting SARS-CoV-2. The work also gives an example of how to organize and process data from ultra-large docking screens.

## Abstract (summary)

**Table:** 

Measurement(s)	equilibrium association constant (KA)
Technology Type(s)	molecular docking by scoring function
Factor Type(s)	Chemical formula and connectivity
Sample Characteristic - Organism	Severe acute respiratory syndrome-related coronavirus
Sample Characteristic - Environment	in-silico

## Background & Summary

The COVID-19 coronavirus pandemic places enormous attention on the application of efficient *in silico* screening of small molecules via molecular docking to expedite the discovery of potential viral inhibitors. As a method, molecular docking uses physical models to produce 3D structures and binding energy estimates for small molecules in binding pockets on proteins, which may interfere with protein activity^1^. By leveraging supercomputing resources, we generated large datasets containing the calculated binding modes and associated estimated binding free-energies energies of over one-billion small molecules to each of several SARS-CoV-2 protein targets^2–4^, as part of a larger effort to make use of supercomputing to accelerate drug discovery while also increasing our understanding of the biophysical behavior of several SARS-CoV-2 proteins^5^. This expansive virtual screening campaign provides an opportunity to provide a comprehensive view of the relevant chemical space and its interactions with key coronavirus proteins. This manuscript compliments our raw data releases and provides details of the methods to generate the dataset and output formats for this dataset to facilitate further analysis.

Because of the approximations used in designing scoring functions, docking is most useful in the initial hit identification phase of the drug discovery pipeline^6^. Hits identified from docking provide inputs including common substructures (pharmacophores), novel chemical scaffolds, and molecular structures suitable for subsequent optimization by rational design. A critical review of the effectiveness of hit generation via docking noted a median hit rate of 13%, much better than random screening (whose hit is approximately 0.1%)^7^. Hit rates can vary between protein targets due to a combination of the approximate nature of scoring functions and protein ‘druggability’. Relevant to the first target present in this dataset, a recent comparison between multiple scoring functions^8^ showed that AutoDock^9^ identified a ligand pose within 2 Ang. RMSD of its crystal for only 40% of 193 crystal structures compared. Of those predicted correctly, the best scoring pose coincided with the crystal 75% of the time.

**Fig. 1:**
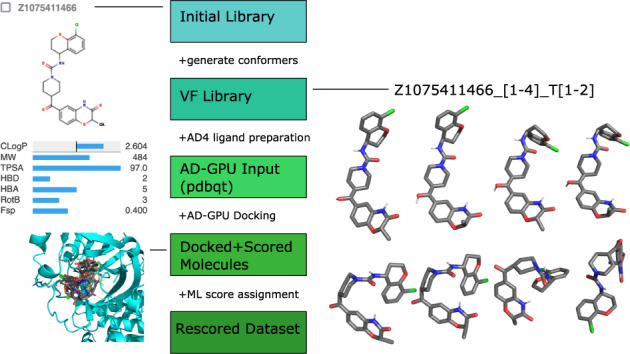
Workflow for creation of docked, scored molecule dataset. Illustrations show: (top left) examples of the initial molecule structures and information available from Enamine^27^, (center right) alternative generated molecular structures from Virtual Flow (VF)^18^, and (lower left) docked geometries. Virtual Flow’s generated geometries are labeled by two additional numbers. The first number enumerates stereocenters and ring puckering variations. The second tries to enumerate tautomerization states. In the example here, T1 and T2 differ by re-interpreting a ketone oxygen as an alcohol–adding double-bonds and removing hydrogens from carbons in the central ring. The inputs are then passed to ligand preparation routines for AutoDock4 (AD4)^9^, which shares input requirements with AutoDock-GPU (AD-GPU)^15^. The docking results can then be rescored with machine learning (ML) techniques.

Typical virtual screening studies^10–12^ aimed at identifying compounds to modulate protein function and disease progression have tended to make use of libraries containing on the order of hundreds of thousands to millions of molecules. Currently, large scale efforts are limited primarily by available computing power, with large catalogs of synthetically available compounds and relatively high hit rates for computational drug discovery^13^. Recent exceptions to this paradigm have begun to emerge, docking hundreds of millions of molecules against single protein targets^4,14^. Here we provide a data release description for docking results for over a billion compounds from the 2018 Enamine REAL dataset against six different protein structures encompassing five different SARS-CoV-2 associated targets in a serial “gigadocking” campaign utilizing GPU-accelerated docking software. The docking process followed for these billion molecule libraries explicitly calculates the location of each (flexible) ligand relative to each target protein’s (rigid) binding site (Fig. 1). This provides an estimated score for the binding free-energy of the predicted complex using well-known, currently established, docking protocols implemented in AutoDock-GPU^15,16^.

This data will be useful both to compare the accuracy of new methods as well as to understand the limitations of established docking protocols. Since the docking dataset presented here covers an unprecedented range of chemical space for proteins that have elicited significant experimental attention, it represents a unique opportunity to compare with both experimental structures and binding affinities. Such comparisons together with rescoring docked poses might yield insights beyond those measured here, similar to rescoring efforts^17^ on the large VirtualFlow dataset^18^. The dataset may also be employed to improve current understanding of non-specific binding^19^. Further, owing to the vast chemical space represented here and the targets selected, the docking scores and poses may serve as a useful sample dataset for those interested in applying machine-learning technologies to analyzing protein-ligand datasets.

The SARS-CoV-2 proteins targeted using gigadocking were: (i) Main protease (Mpro), which has a important role in mediating viral replication and transcription^20^; (ii) Papain-like protease (PLpro), which is essential to cleave viral polyproteins and assemble the replicase-transcriptase complex^21^; (iii) Endoribonuclease (Nsp15), a uridine specific endoribonuclease, that processes viral RNA to evade recognition by host defense systems^22,23^; (iv) RNA-dependent RNA polymerase (RdRp) that regulates viral replication^24^; (v) the Spike protein, which is recognized by the ACE2 receptor to initiate membrane fusion that triggers cellular infection^25,26^.

## Methods

The molecules described in the study are a subset of the 2018 Enamine REAL database^27^, prepared by VirtualFlow^18^. Molecules within the REAL database are readily synthesizable compounds that can be made on-demand using in-house validated procedures. For consumers of this dataset, it is important to note that Enamine offers a newer version of its database that is preferred for new projects^27^. The chemical structures of the 1.4 billion compounds used for docking are identical to those inflated by VirtualFlow and made available at the beginning of the pandemic^18^, as these pdbqt files were used in the initial screen. All compounds were docked against binding sites of six SARS-CoV-2 proteins, selected for their potential as drug targets (Fig. 1).

Both ligand and protein structures were converted to AutoDock-compatible input formats (map files and pdbqt) using AutoDock Tools^9^. While the existing VirtualFlow ligand structures were provided in a AutoDock compliant format, inspection of pre-assigned partial charges in the existing library showed deviations from AutoDock standards. As a result all ligands were re-processed using AutoDock Tools for proper partial charge assignment. These inputs for AutoDock-GPU are included in the dataset. Docking was carried out using the Summit supercomputer and Google Cloud high performance computing resources using the AutoDock-GPU program as it stood June 2020^15,16^. AutoDock-GPU computed 20 independent structures per small molecule ligand-protein combination using the Solis Wets search method^15^. In addition to the AutoDock estimated binding affinity, we also report the rescoring results for RFScore v3^28^ and DUD-E^29^ machine-learned rescoring models.

### Data generation protocol

1. Protein map files were prepared using AutoDock Tools^9^ with input from the entire team. The map files dictate the search space for where ligand-protein interactions may occur, and are chosen around selected regions of interest for each protein, such as enzyme active sites. In this way, the experimental design favored competitive inhibitors that could interfere with the protein-protein interface, rather than allosteric binders to cryptic sites which are only uncovered by additional simulation^30^.

#### MPro

The active site of the MPro protein has previously been shown to have a large amount of conformational plasticity^31^; to account for this two MPro structures were chosen for docking. The two structures chosen for docking are found in the protein databank^32^, with accession codes 5R84^33^ and 6WQF^31^. The 6WQF structure was chosen as one starting point as it represented a “thermalized” structure, having been solved at high temperature. The 5R84 structure was selected as an alternative structure with a relatively accessible active site due to the bound ligand. These two structures were aligned to the 5R84 structure, with the selected region for docking chosen to be located at (−8, 6, 8) relative to C_*α*_ on Pro39. This box center was chosen to include bound ligands from existing crystal structures near the active site while minimizing the box size to maximize throughput. Thus, the input grid for AutoDock-GPU was a 65 × 65 × 65 gridpoint cube, with spacing between gridpoints of 0.375 Å.

#### PLPro

The initial structure for the papain-like protease (PLPro) was taken from the protein databank entry 7JIR^34^ with ligands and Zn2+ removed. Two crystallographic waters located near Tyr273 and Thr301 were retained from the original crystal structure. An additional strongly bound water at Asp164 was considered, but not retained during docking. To properly orient the crystallographic waters, a short energy minimization (1000 steps) was performed using the CHARMM36 force-field^35^ in NAMD2.14^36^. The docking search box, following the same protocol noted above for MPro, was constructed with the center taken to be located at (51.51, 32.16, −0.55) in the PDBQT file, which corresponded to the center of the original ligand present in the crystal structure. The C111S mutation was preserved in our gigadocking screen.

#### RdRp

An initial structure for the SARS-CoV2 RNA-Dependent RNA Polymerase (RdRp) was obtained from the protein databank (6YYT)^24^; however, the initial structure lacked the reasonable placement of Mg2+, Zn2+, and ATP within its active site. To correct for this deficiency a series (1,000) of alternative models derived from distance restraints taken from a similar RdRp found in the Hepatitis C virus (4WTD)^37^ was generated using the Rosetta homology modeling package^38,39^ and the top performing, as determined by the Rosetta energy score, was selected for alignment with the PDB code 4WTD to refine the placement of ATP. Following the addition of ATP and ions, an energy minimization/structure optimization calculation, performed with a gradient descent and with a convergence target of 0.0001 RMS kcal/mol/Å2 using MOE2019^40^ with the Amber14:EHT^41^ force-field, was performed. To ensure the ions did not drift too far during the minimization, harmonic tethers were used to restrain ion positions to within 0.5 Å. Additionally, the MOE option to optimize OH positioning was also used. The final relaxed structure was used for the docking and the docking box center was taken as (93.88, 83.08, 97.29), near the center of the RNA binding site.

#### NSP15

For Non-structural protein 15 (NSP15), the initial structural model used for docking was derived from the crystal structure (PDB 6WLC) of SARS-CoV2 NSP15 Endoribonuclease in complex with substrate Uridine-5′-monophosphate^42^. The docking search box was centered near Tyr343 (93.93, −16.14, −30.06) with identical input grid dimensions as in the previously described proteins, a 65 × 65 × 65 gridpoint cube, with 0.375 Å spacing between gridpoints. The center and size of the box were chosen to include the enzyme active site (i.e., residues around the bound ligand).

#### Spike protein

The protein structure (PDB 6M0J) of the SARS-CoV2 Spike protein subunit S1 in complex with Angiotensin-converting enzyme 2 (ACE2)^26^ was used for docking. The ACE2 and coordinating ions were removed. The box was centered at XYZ coordinate of −34.968, 25.439, 3.367 with a 64 × 64 × 59 input grid and 0.375 Å gridspacing. In this design, bound ligands would be predicted to interfere with the Spike-ACE2 binding interface.

Grids for C, N, OA, HD, A, NA, F, SA, S, Cl, P, I, Br, and Fe atomtypes were generated using AutoGrid 4 for all proteins. The grids themselves were generated with the smooth parameter set at 0.5, and the distance-dependent dielectric factor set at −0.1465^43^, as is the default for AutoDock4^9^.

2. The pre-computed 3D structures of the Enamine 2018 REAL library provided by VirtualFlow^4^ were pre-processed, using the prepare_ligand.py tool from the AutoDock Tools suite^9^, to re-assign partial charges to AutoDock standards. Following charge re-assignment, the ligand library was split into tarball archives. Following the initial docking of MPro 6WQF, preliminary analysis of docked poses found several ‘broken’ ligands (about 0.1%). A full description is present in the section on ‘Data Integrity’. Invalid molecules were removed from the input library when screening all docking targets other than MPro.
3. Docked compounds moved to Google Cloud Storage were aggregated into groups and sequentially re-numbered to make batch sizes around 1024 molecules. This new batch size decreased the docking time per batch to around 10 minutes.
4. Docking was carried out on the six systems using slightly different workflows based on the available high performance computing resources:
  - MPro_5R84 used a Fireworks-based workflow run on Summit and Marble
  - MPro_6WQF, NSP15_6WLC and Spike_6M0J used launchad on Summit. (see code availability below)
  - PLPro_7JIR and RDRP used launchad customized for running using on-demand compute nodes on Google Cloud
  Additional workflow and analysis tools developed for MPro docking are described in Ref. ^44^.
5. For runs on Summit, docked/nn.pq files were created by post-processing docking log-files using pymapreduce (see code availability below) from the Rhea and Andes systems at the Oak Ridge Leadership Computing Facility (OLCF). Then rescoring (generating scored/nn.pq) was accomplished by running the pmake parallel make manager^45^ with rescore.py (present in the secondary dataset). This version parallelized over single input files. The yaml files in the top-level directory of the primary dataset document its run inputs.
6. For runs on Google Cloud, docked/nn.pq files were output directly by launchad and rescoring using launchad’s rescore.py function to create scored/nn.pq outputs.

Note that the batch sizes after re-scoring also differ between Summit and Google Cloud runs. For Summit, all the compounds in docked/nn.pq files correspond to scores in scored/nn.pq files (same nn). However, for Google Cloud runs, the compounds in docked/nn.pq correspond to scores in scored/$(nn/4).pq. This difference in batch sizes for docked compound outputs explains why there are 4 times more docked/nn.pq files for the Google Cloud runs.

The highest score from each molecule is the usual criterion used to rank the data. Even though 20 conformers were generated by the docking output, only the best 3 docked geometries for each molecule were saved in this dataset. Others can always be re-generated by docking again. Every record contains one molecule with all three scores. They are numbered in order of AutoDock “score”^15^. For PLPro_7JIR, the “best 3” AutoDock scores were used. For all other proteins, one pose from each of the “best 3” AutoDock pose clusters was used. AutoDock performs this clustering based on ligand atom RMSD^9^. The pose clusters recognize that docked poses often find the same energy minimum. So, using one result from each cluster increases the variability between bound ligand conformations.

## Data Records

The outputs of this work are divided into primary^46^ (output) and secondary^47^ (derived, summary, and comparison) data-tables.

### Primary data

Primary data include data describing the input molecules, as well as two datasets for each docked protein–one containing full molecular structures, and another containing only docking scores. Each dataset is split into multiple numbered files in Apache Parquet format^48^.

To describe input molecules, we include SMILES strings^49^ in files named Enamine_2018/smiles0.pq… Enamine_2018/smiles999.pq. Each record of each parquet file has both a name and a SMILES string^49^. For example, the molecule with name Z979400128_1_T1 is defined by the SMILES string beginning with O = C(CN1C( = O)CCC1 = O)N1CCN(CC1)C( = O)[C@@H]1CCCN… .

As for the data on each docking target, we provide an individual directory for each target with a high-level README describing the data layout. Within this directory is also where the input pdbqt and AutoDock compatible grid data are stored, with the latter compressed for efficiency. Although the number of files and batch sizes vary from target to target based on which computing resource carried out the docking, the data layouts are consistent between all targets.

Within each protein target, the docked dataset contains multiple numbered files in Apache Parquet format^48^. Each individual file represents a batch of approximately 150,000–500,000 ligands docked to a target, with the following typical columns:

- name: extended molecule name, formatted as enamine-id_conformer_id. For example PV-001921702752_1_T1, has conformer id 1_T1.
- atoms: number of atoms in this molecule*
- tors: number of rotatable bonds*
- conf, conf2, conf3: pdbqt-formatted docked poses
- score, score2, score3: AD-GPU docking scores corresponding to the poses above.

The conformers and scores are presented in ranked order (lowest energy first). For all targets except PLPro-7JIR, the docked conformers (conf1-3) represent the best pose from a group (determined by AutoDock’s RMSD-based ligand clustering^9^). For PLPro-7JIR, the conformers were not clustered, leading to overlapping poses in its dataset. Entries atoms and tors are starred (*) above because data for PLPro and RDRP targets lack these columns. They can be easily recovered from the text of the conf column by counting the number of ATOM and BRANCH lines. Source code that accomplishes this is included in the file dash/data/add_counts.py (from secondary data). The method does not rely on parsing TORSDOF from the conformer’s pdbqt, as there are a few molecules for which TORSDOF is an over-estimate.

The scored dataset contains only score values:

- name, atoms, tors, score, score2, score3: same as for dataset above
- vs_dude_v2, vs_dude_v22, vs_dude_v23: Virtual-Score DUD-E score based on v2 descriptors for molecule poses 1, 2, and 3.
- rf3, rf32, rf33: Random Forest v3 (RF3) score for molecule poses 1, 2, and 3.

### Secondary data

We have generated a variety of smaller, secondary datasets in the process of analyzing the docking results. They are made available for direct download at ref. ^47^. These include:

- Top-10,000 lists using multiple scoring criteria (see Table 1).

**Table 1:** Scoring Cutoffs for Single and Intersection Lists.

List	Cutoff Percentile	Cutoff Score 1	Cutoff 2	intersection size
MPro 5R84				
AutoDock-GPU (AD)	99.999	−13.220		11,754
RF3 (RF)	99.999	8.032		10,168
AD-RF	99.874	−11.786	7.768	15,843
VS-RF	99.978	6.714	7.874	14,124
VS-AD	99.866	6.460	−11.668	10,910
MPro 6WQF				
AutoDock-GPU (AD)	99.999	−12.865		10,167
RF3 (RF)	99.999	7.954		12,009
AD-RF	99.903	−11.474	7.722	10,731
VS-RF	99.984	6.618	7.825	12,745
VS-AD	99.891	6.369	−11.332	10,984
PLPro 7JIR				
AutoDock-GPU (AD)	99.999	−15.261		11,804
RF3 (RF)	99.999	8.176		10,530
AD-RF	99.976	−14.062	7.961	10,153
VS-RF	99.813	6.349	7.800	12,128
VS-AD	99.865	6.359	−13.327	11,979
NSP15 6WLC				
AutoDock-GPU (AD)	99.999	−13.534		11,593
RF3 (RF)	99.999	7.984		11,211
AD-RF	99.719	−11.551	7.597	13,795
VS-RF	99.919	6.194	7.701	15,750
VS-AD	99.823	6.161	−11.663	10,332
Spike 6M0J				
AutoDock-GPU (AD)	99.999	−11.739		10,277
RF3 (RF)	99.999	7.970		12,005
AD-RF	99.766	−10.078	7.459	12,364
VS-RF	99.576	6.106	7.352	12,692
VS-AD	99.895	6.148	−10.330	11,869
RDRP				
AutoDock-GPU (AD)	99.999	−12.435		11,679
RF3 (RF)	99.999	8.021		16,604
AD-RF	99.905	−11.032	7.722	13,462
VS-RF	99.905	6.329	7.722	11,858
VS-AD	99.871	6.304	−10.813	10,271

Intersections are taken as the logical ‘and’ of top-n molecules from both functions listed. These are output by plot_score_hist.py and used for selection in sublists.py.
- Source code for working with the dataset (including write_confs.py for extracting top-10k lists).
- Summary histograms to describe the docking score distribution. (see Fig. 3)

**Fig. 2:**
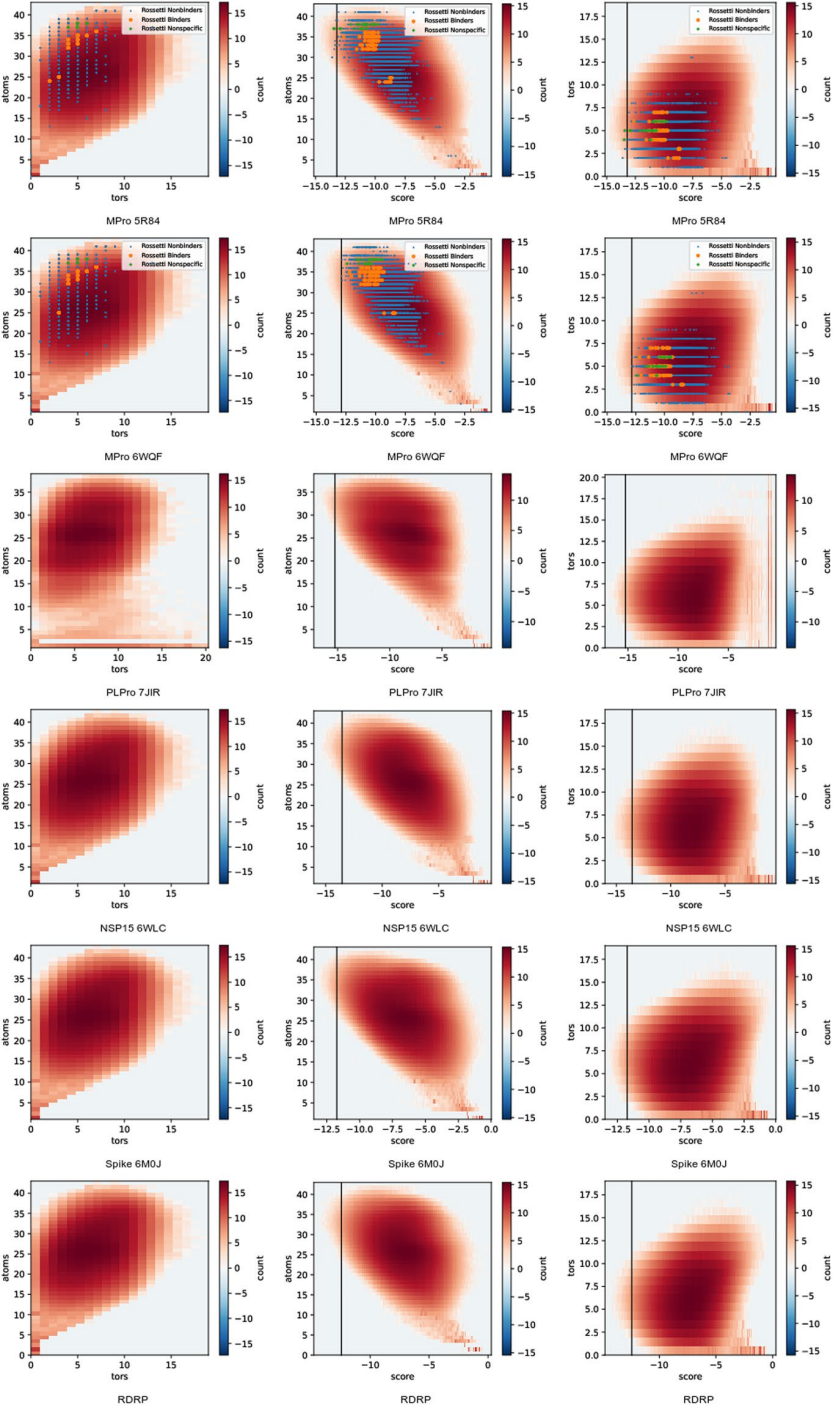
Molecule atom and torsion count 2D histograms, plotted on a natural logarithmic scale to emphasize the tails of the distribution. Columns contain, from left to right, (atoms,tors), (atoms,score) and (tors,score). Score refers to AutoDock-GPU’s score. Atoms refers to the count of heavy atoms plus polar hydrogens present in the pdbqt file used for docking. Tors refers to the count of torsion degrees of freedom marked in that pdbqt. Marked points show our data values for all compounds listed in ref. ^50^ (pooling all isomers and geometries).

**Fig. 3:**
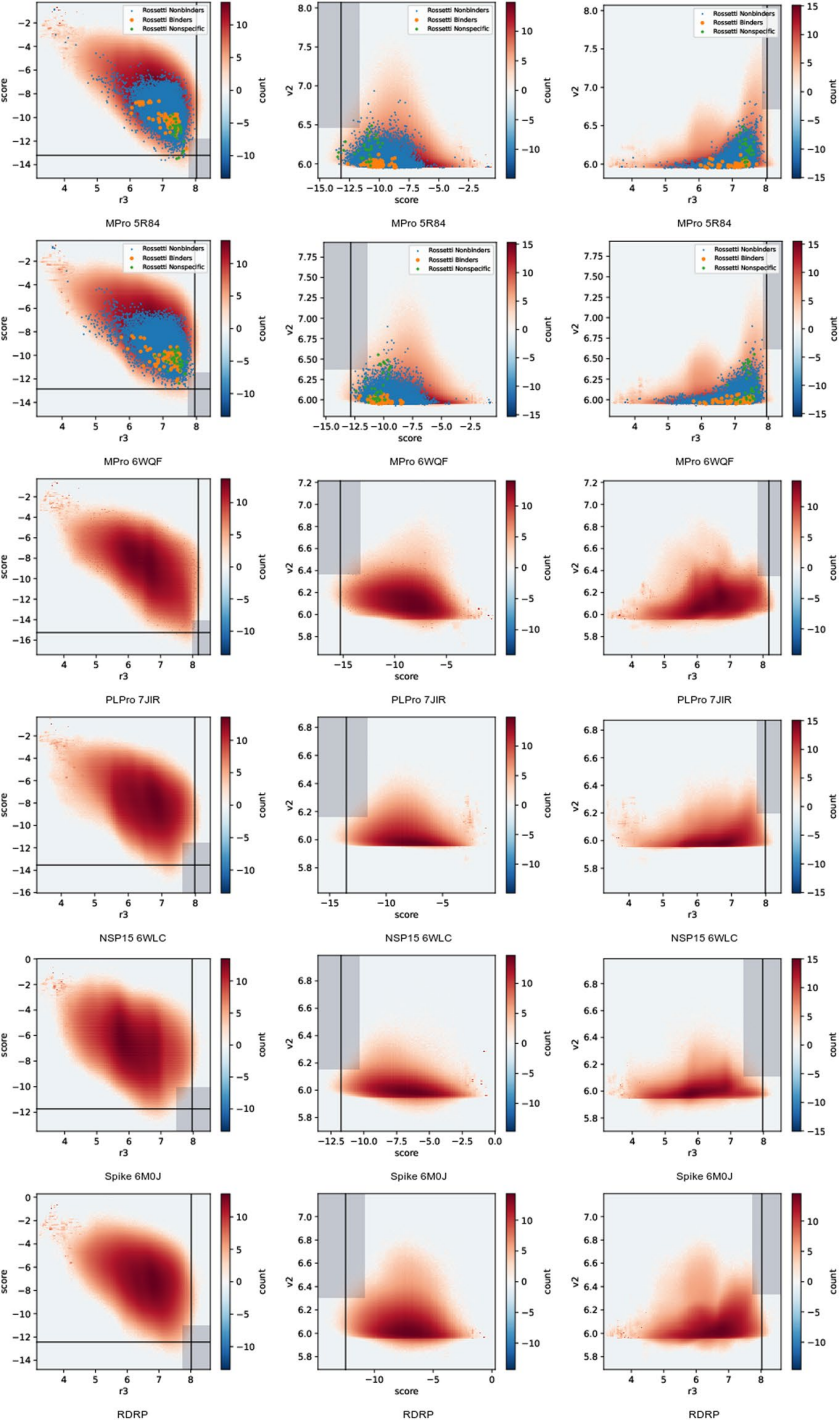
Docking score 2D histograms, plotted on a logarithmic scale to emphasize the tails of the distribution. Columns contain, from left to right, (score,r3), (v2,r3) and (v2,score). Score refers to AutoDock-GPU’s score, r3 and v2 refer to re-scoring of each docked pose based on random-forest functions parameterized from RF3 and Virtual-Score DUD-E, respectively. Lines and boxes mark the score cutoffs used for creating top-10k lists based on single and joint-score criteria, respectively. Marked points show our data values for all compounds listed in ref. ^50^ (pooling all isomers and geometries).
- Selected compounds (for MPro) corresponding to experimental measurements from ref. ^50^.

Top-10,000 lists were produced for all dockings. These lists are in the same parquet format as the docked and scored lists above, but contain the union of docked and scored data. Every record has both scores and structures for the top 3 ligand conformational poses. Our methodology for creating these lists was to compute percentiles based on the score histograms (Fig. 3), find the cutoff score corresponding to that percentile, and then retain all molecules from the dataset above the cutoff. This created list sizes that differed slightly from 10,000 (Table 1). In cases where two scoring functions are listed, the cutoff criteria required that molecules be in the top N^*th*^ percentile for both scores. Cutoffs were determined so that the percentile, *N*, was the same (to within a histogram bin width) for both scoring functions. The score cutoffs, percentile ranks, and resulting list sizes are listed in Table 1.

We also created a ‘random’ list containing around 10,000 ligands. The selection criteria used was based on a 10-byte blake2b hash-digest of the molecule’s name, interpreted as a big-endian number modulo 2^31^—1. Molecules with a value less than $\left({2}^{31}-1\right)\frac{10300}{1.3\cdot 1{0}^{9}}$ were included in the random list. This process ensured that the random list would contain the same molecules between dockings, while membership in the random list was testable. The implementation code is in the function dataset.sublists:rand_10k.

Ref. ^50^ carried out experimental measurement of candidate molecules from several large virtual screening experiments on MPro. The rossetti2022.csv file in our summary dataset^47^ collects the Supplementary information tables from that work, and adds annotations for the experimental inhibition measurements present in its figures and main text. The 1148 unique compounds tested there were divided into three sets: nonbinders (1131), hits (13), and nonspecific hits (4). Hits are defined as all compounds whose affinities were reported, excluding nonspecific hits. Nonspecific hits are compounds with reported affinities, but which ref. ^50^ mentioned were nonspecific binders.

Output score histograms are shown in Fig. 3. Lines on these plots mark the cutoff scores for top-N lists based on one scoring function only. Shaded boxes on those plots map the cutoff criteria for lists based on cut-offs from two scoring functions simultaneously. Because the set of all docked molecules represents a very large, random, selection from chemical space, these histograms illustrate chemical space as seen from the receptor. Interestingly, none of the receptors show a distinct cut-off between strong and weak binders. Rather, most of chemical space has an average docking score.

The plotted points on Fig. 3 allow us to validate the usefulness of docking scores for MPro. We are essentially interested in four regions: true positives (hits with good docking scores), true negatives (nonbinders with poor docking scores), false positives (nonbinders with good docking scores), and false negatives (binders with poor docking scores). We see strong enrichment of nonspecific binders in the high-scoring range. It also appears that the Autodock score is better correlated with separating binders from non-binders, and that the wider range of Autodock scores for 6WQF makes this separation more pronounced. The Virtual-Score DUD-E and RF3 scores show a high rate of false positives. It should be noted that the compounds selected for experimental testing were pre-selected using docking or chemical similarity to other experimental hits. We can’t draw immediate conclusions from the wide range of our docking scores for this compound set as a whole, because this may reflect variability in the computationally enumerated chemical states for a given compound.

## Technical Validation

### Data integrity

Parquet data has been stored using the SNAPPY^51^ compression algorithm, which provides an internal checksum to verify data integrity. All data manipulation steps during docking and scoring retained unmodified molecule names within each row of each table. Since molecules were processed in batches of thousands of molecules, lists of batch numbers were compared before and after each batch processing step. Missing batches were re-run until all batches were output. An exception is in the case of MPro 5R84, which used a different batch processing scheme that did not permit identification of missing batches. Despite this effort, total molecule counts vary by about 0.1% between dockings to different targets. These small differences reflect molecules that dropped out due to errors during formatting or docking. Where possible, files named errors.parquet (in the primary dataset) document the types of errors found on parsing scores and conformers from the docking output. These errors are, 0: molecule has no atoms (97%), 1: AutoDock-GPU score is larger than 100 or nan/inf, indicating that the molecule did not end in the docking region (0.5%), 2: coordinate records could not be parsed (0.5%), and 3: atoms overlapped (2%). Ligands lacking atoms or overlapping significantly were traced back to pre-existing errors in the pdbqt files input to the docking from the original VirtualFlow dataset^18^.

Histograms of the number of heavy plus polar hydrogen atoms and torsion degrees of freedom in the output molecules are shown in Fig. 2. Both atom and torsion counts fall into a relatively narrow range around 25 atoms and 10 torsions because the input database used was built to represent drug-like molecules. The number of molecules with small atom counts is somewhat surprising. Figure 2 also includes joint histograms with AD-GPU score. As expected, there is a positive correlation between atoms and number of torsion degrees of freedom. There is also a correlation between atom count and docking score, with more atoms tending to enable more favorable (negative) scores. There is not a clear correlation between torsion number and docking score. These trends are chemically reasonable and show consistent trends between all protein targets.

### Cross-validation

**Fig. 4:**
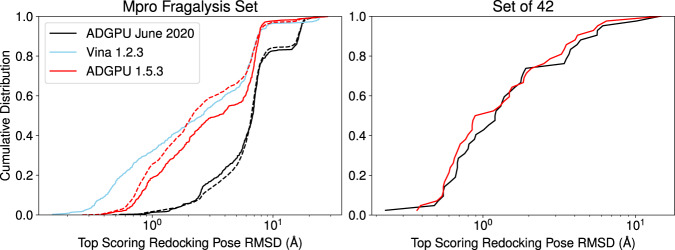
Redocking RMSD distribution against the Mpro Fragalysis dataset (left) and the AutoDock-GPU set of 42 (right), measuring the deviation between a predicted docking pose and an actual crystal structure. The fragalysis dataset (left) docks 426 ligands where bound crystal structures are available from the Diamond dataset, and compared the displacement for only the top-scoring pose from AutoDock Vina (blue)^52^, the current version of AutoDock-GPU (red, 1.5.3)^15^ and the version of AutoDock-GPU used to generate the dataset (black, June 2020). Beyond the top poses, the distribution for all 20 predicted poses for both versions of AutoDock-GPU are given as a dashed line. The set of 42 (right) similarly compares the cumulative RMSD distribution generated after redocking.

Prior to the beginning of the docking campaign, we evaluated AutoDock-GPU performance for a set of 42 protein-ligand interactions with known structure as a sanity check for methodological consistency. The results for the AutoDock-GPU CUDA-port were comparable to prior OpenCL-driven implementations, and this gave us the confidence to attempt the first billion compound docking campaign in June 2020. The version of AutoDock-GPU used in the docking described above still performs well on the small set of 42 test receptors and ligands when compared to newer versions of AutoDock-GPU (Fig. 4), with a small median deviation from the crystal stucture (1.18 Å). This is comparable to recent versions of AutoDock Vina^52,53^, which uses a different scoring function, and newer versions of AutoDock-GPU^15,16^.

A more stringent test for docking quality may be using the same docking methods against crystal structures for MPro with bound ligands. The Fragalysis dataset^54^ from the Diamond lightsource contains a few hundred bound ligands to the MPro structure for comparison. The state of docking programs has advanced considerably as part of a wider drug discovery effort driven by the pandemic. The typical RMSDs for redocking the bound ligands within the fragalysis dataset using the older version for AutoDock-GPU are much larger, with a median redocking RMSD of 6.6 Å. By comparison, the median redocking RMSD can be as low as 2.1–3 Å (Fig. 4) for docking codes newer than the AutoDock-GPU version used to generate this dataset.

Clearly, the accuracy for docking methods has been improved and refined since June 2020, and billion-compound scale dockings should use these newer methods. However 13% of top scoring redocked poses are within 3 Å RMSD of the crystallographically determined binding pose within this dataset. While the RMSD can be quite large, the contacts made between protein and ligand are largely similar, allowing rescoring techniques to be used. The median center of geometry difference between the docked and crystallographic ligand poses are within 2 Å of one another using AutoDock-GPU versions from 2020. Recent implementations improve the median center of geometry error to just below 1 Å.

## Usage Notes

**Fig. 5:**
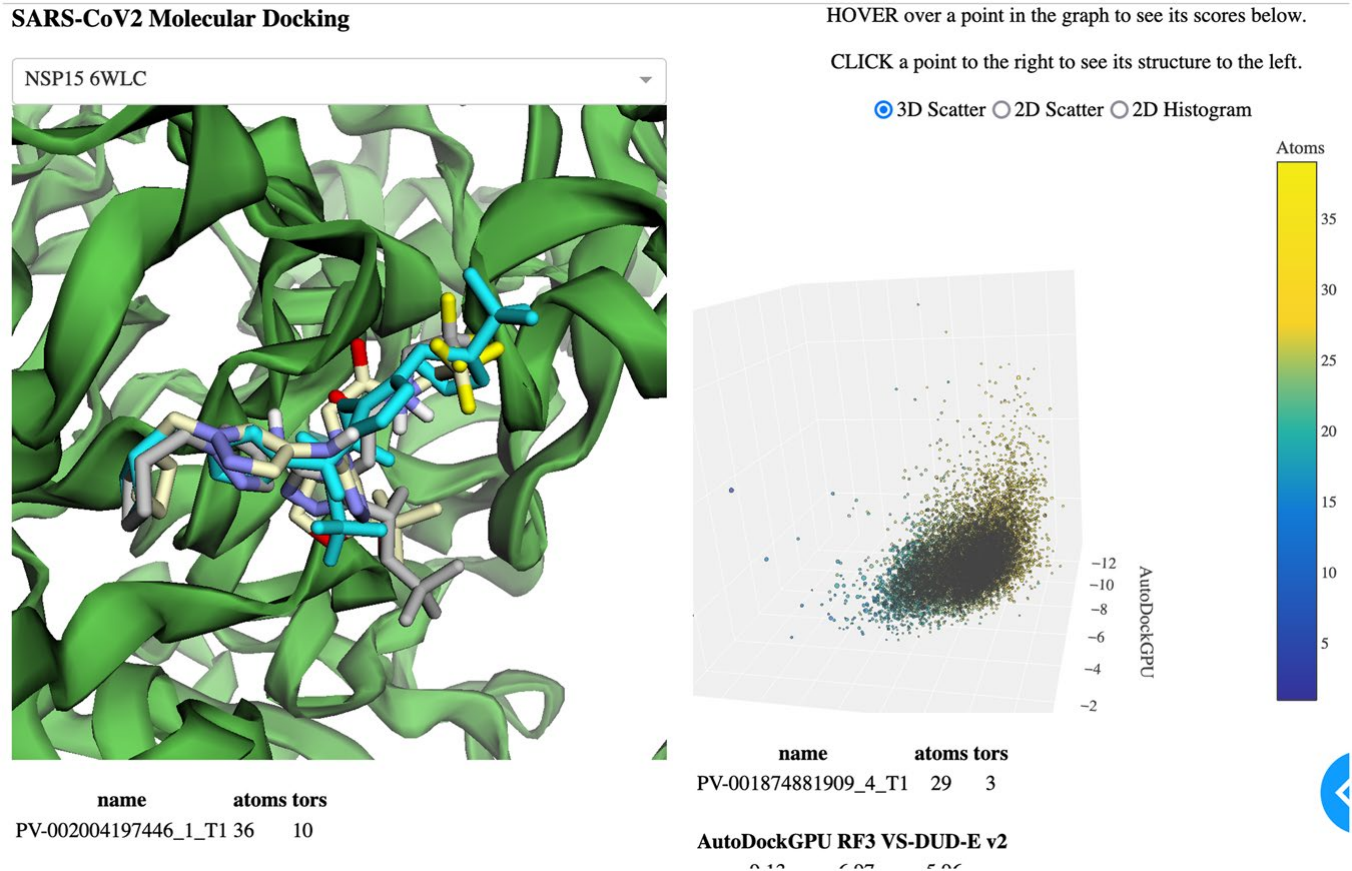
Static image of molecular viewer. Each point on the scatter-plot of AD-GPU, RF3, and VS-DUDE-v2 scores represents one molecule. Selecting the scored point shows its three docked poses in the 3D structure at left, along with information boxes showing the molecule name and numerical data below.

The dataset release contains two components–the main dataset^46^, and the summary^47^. A python-dash^55^ viewer program is included as part of the git repository (Fig. 5). For convenience, protein structure files exist in both places. All ligand data files are packaged in the Apache Parquet^48^ file format. They can be opened using fastparquet^56^, as well as many data science libraries such as pandas^57^.

The dockingdata/src directory contains a set of scripts for loading data and running map-reduce operations, like computing histograms. The dash directory contains a viewer that can be run by executing the program and connecting to it from a web browser. It is straightforward to view alternative subsets of the data by directing it to load different input files.

**Fig. 6:**
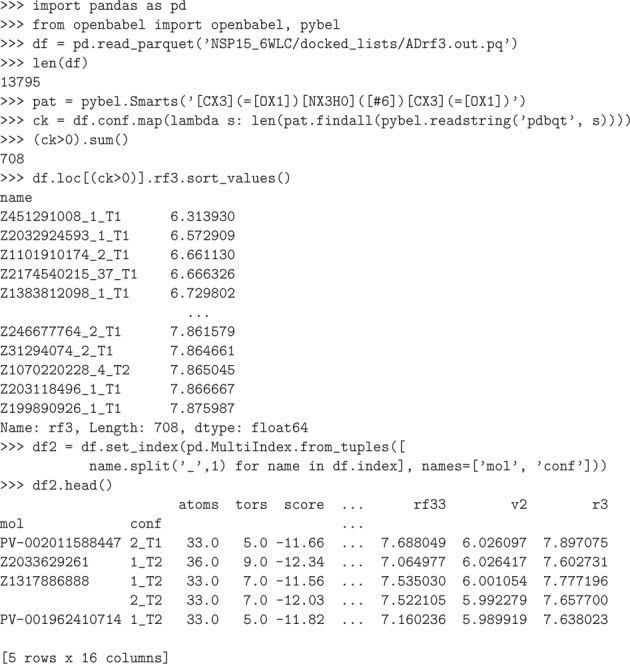
Use of pandas^57^ and pybel^58^ to select hit molecules containing dicarboximide functional groups. After loading a top-N dataset, molecules are parsed by openbabel^59^ and then a SMARTS^60^ pattern search is applied to each row. The last line of the program shows an example of changing the index of the dataset to separate molecules and enumerated rotamers.

Following the example scripts present, researchers can quickly run simple queries on the top-N subset lists. Figure 6 gives a code listing showing how to select hits containing dicarboximide functional groups from the set of molecules with high AutoDock and rf3 scores.

## Data Availability

The primary version of AutoDock used to generate the primary dataset is available from https://github.com/jvermaas/autodock-gpu. As noted in Fig. 4, this is not recommended for new docking calculations. Instead, new projects should use current versions of AutoDock-GPU^15^ such as 1.5.3, available from https://github.com/ccsb-scripps/AutoDock-GPU. To generate the Vina data for Fig. 4, we used Vina 1.2.3^52^ from https://github.com/ccsb-scripps/AutoDock-Vina. As described in the ‘Data Generation Protocol’ section, several custom software packages were developed and used in this project, including 1. https://code.ornl.gov/99R/launchad/-/tags/v1.2 2. https://code.ornl.gov/99R/pymapreduce 3. https://code.ornl.gov/99R/pmake^45^ 4. https://github.com/frobnitzem/mpi_list 5. https://github.com/frobnitzem/sars_docking^47^
